# Gene-environment interaction counterbalances social impairment in mouse models of autism

**DOI:** 10.1038/s41598-019-47680-w

**Published:** 2019-08-07

**Authors:** Ji-Woon Kim, Kwanghoon Park, Ri Jin Kang, Edson Luck Gonzales, Hyun Ah Oh, Hana Seung, Mee Jung Ko, Jae Hoon Cheong, ChiHye Chung, Chan Young Shin

**Affiliations:** 1 Department of Pharmacology and Department of Advanced Translational Medicine, School of Medicine, Konkuk University, 120 Neungdong-ro, Gwangjin-gu, Seoul, 05029 South Korea; 2 Department of Biological Sciences, Konkuk University, 120 Neungdong-ro, Gwangjin-gu, Seoul, 05029 South Korea; 3 Uimyung Research Institute for Neuroscience, Department of Pharmacy, Sahmyook University, 815 Hwarangro, Nowon-gu, Seoul, 01795 South Korea; 4 NeuroVenti, Inc. and TriNeuro Inc., 120 Neungdong-ro, Gwangjin-gu, Seoul, 05029 South Korea

**Keywords:** Social behaviour, Autism spectrum disorders

## Abstract

Autism spectrum disorder (ASD) is a neurodevelopmental disorder characterized by social communication deficits and repetitive/restricted behaviors. Although gene-environment interactions may explain the heterogeneous etiology of ASD, it is still largely unknown how the gene-environment interaction affects behavioral symptoms and pathophysiology in ASD. To address these questions, we used *Cntnap2* knockout mice (genetic factor, G) exposed to valproic acid during embryonic development (environmental factor, E) as a gene-environment interaction (G × E) model. Paradoxically, the social deficits observed in the respective G and E models were improved in the G × E model; however, the high seizure susceptibility was more severe in the G × E -model than in the G and E models. Repetitive self-grooming and hyperactivity did not differ among the three models. The amplitudes of miniature excitatory postsynaptic currents in layer 2/3 pyramidal neurons of the medial prefrontal cortex were aberrant and similar in the G × E model when compared to the control group. Our findings suggest that the interaction of two risk factors does not always aggravate ASD symptoms but can also alleviate them, which may be key to understanding individual differences in behavioral phenotypes and symptom intensity.

## Introduction

Autism spectrum disorder (ASD) is a neurodevelopmental disorder characterized by social communication deficits and restricted/repetitive behaviors before 3 years of age^1^. Etiological heterogeneity and complex behavioral symptoms hamper understanding of ASD. Over 1000 genetic factors^2^ and various environmental factors such as valproic acid (VPA), maternal immune activation, and paternal age can induce ASD^3,4^. A multitude of genome-wide association studies have been conducted to identify a common target gene but have not been successful^5–7^, indicating that the etiology of ASD may not be explained by one common genetic factor. Instead, recent studies have shown that autistic symptoms can be caused or exacerbated by the interaction of genetic and environmental risk factors^8,9^, suggesting that gene-environment interaction may be a mechanism underlying the etiology of ASD. Yet, how the gene-environment interaction affects complex behavioral symptoms and pathophysiology in ASD remains largely unknown.

To date, one of the most important hypotheses to explain the pathophysiological mechanism of ASD is excitatory-inhibitory (E/I) imbalance^10,11^. E/I imbalance can result from abnormalities in excitatory or inhibitory neural structure and/or function^11,12^. The reduced or increased synaptic transmission may cause overprocessing (noise)^10^ and misprocessing (disconnection)^11^ of the transmission, respectively. The impaired transmission may cause connectivity problems in the neural circuits and thereby may lead to neurodevelopmental disorders such as ASD^10,11,13^. The impact of E/I imbalance may depend on the affected brain region. The prefrontal cortex (PFC; or medial prefrontal cortex, mPFC) is a very well-known brain region mediating social behavior^14^. Indeed, neural activity in the mPFC is increased during social behaviors^15,16^. An optogenetic study showed that over-activation of excitatory neurons in the PFC induces abnormal social behaviors^17^, recapitulating the effect of E/I imbalance in the mPFC as an inducer of social impairment.

In our previous study, contactin-associated-protein-like 2 knock-out mice (*Cntnap2* KO, a genetic risk factor model) and mice exposed to VPA during embryonic development (VPA mice, an environmental risk factor model) displayed reduced and increased miniature excitatory postsynaptic currents (mEPSCs) in layer 2/3 pyramidal neurons of the mPFC, respectively, possibly because of aberrant expression of glutamate receptors^18^. Of note, although these two models show opposite neural transmission impairment in the mPFC, these mice show similar behavioral symptoms such as social deficits, repetitive behavior, hyperactivity, and seizure susceptibility^18–20^. Thus, it will be tantalizing to study how the interaction of these factors would affect behavioral abnormalities and the pathophysiology of the respective models.

To investigate the effect of gene-environment interaction, we injected VPA into pregnant *Cntnap2* heterozygous female mice that had been mated with *Cntnap2* heterozygous male mice on embryonic day 10.5 (E10.5) and investigated the autism-like behavioral phenotypes in the resulting offspring. We also investigated neural transmission changes using whole-cell patch clamp in layer 2/3 pyramidal neurons of the mPFC. We found that the interaction of *Cntnap2* KO and prenatal exposure to VPA changed the phenotypes observed in the respective single-factor models. Remarkably, the interaction model showed improvement in social deficits in comparison with the single-factor models, whereas the levels of repetitive-grooming and locomotor-activity in the interaction group were comparable to those in the single-factor models. The interaction also increased seizure susceptibility in comparison with that of the single-factor models. The depressed and enhanced excitatory transmission in the mPFC of *Cntnap2* KO and VPA-exposed mice, respectively, was rescued by the interaction of *Cntnap2* KO and prenatal exposure to VPA. This study provides crucial experimental evidence on how the interplay of two risk factors contributes to phenotypic complexity and on the possible role of E/I imbalance in the modulation of social impairment in ASD.

## Results

### Social impairment is restored by the interplay of *Cntnap2* KO and prenatal VPA exposure

**Figure 1:**
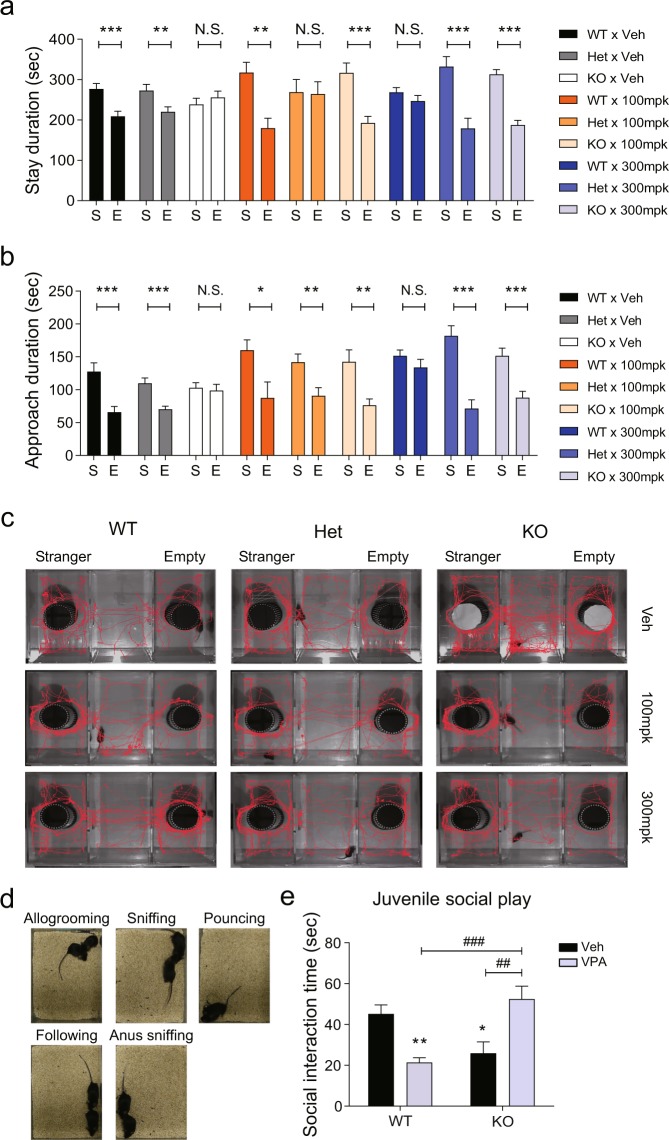
Social deficits caused by *Cntnap2* knockout and valproic acid exposure are normalized by the interaction of these factors. (**a–c**) Three-chamber social interaction test. (**a**) Stay duration in the indicated compartments. (**b**) Approach duration near either the cage with a conspecific or the empty cage. (**c**) Representative movement traces of subject mice. An unpaired *t*-test or Mann-Whitney test was performed to determine significant differences. WT × Veh, n = 18; Het × Veh, n = 35; KO × Veh, n = 25; WT × 100, n = 8; Het × 100, n = 10; KO × 100, n = 17; WT × 300, n = 11; Het × 300, n = 19; KO × 300, n = 16. (**d**,**e**) Juvenile social play (P23–26). (**d**) Measured social behaviors during the test, (**e**) Cumulative social interaction time. Two-way ANOVA followed by Bonferroni multiple comparison test was performed to determine between-group differences. WT × Veh, n = 9; KO × Veh, n = 8; WT × VPA (300 mg/kg), n = 9; KO × VPA (300 mg/kg), n = 9. In **e**, asterisk (*) shows the results of statistical comparison between WT × Veh and the indicated group; *, *p* < 0.05, **, *p* < 0.01. Sharp (#) shows the results of statistical comparison between the indicated groups; *##, p* < 0.01, ###, *p* < 0.001. In all bar graphs, data are the mean ± S.E.M. Abbreviations: S, compartment or cage with a novel conspecific; E, compartment or cage with no conspecific, WT, *Cntnap*2 WT mice; KO, *Cntnap*2 KO mice; Het, heterozygous mice; 100 mpk, 100 mg/kg of VPA; 300 mpk, 300 mg/kg of VPA; VPA, treatment with valproic acid at E10.5; Veh, treatment with phosphate-buffered saline at E10.5.

To investigate gene-environment interaction, we injected phosphate-buffered-saline (PBS) or VPA (100 or 300 mg/kg) into pregnant *Cntnap2* heterozygous female mice that had been mated with *Cntnap2* heterozygous male mice on E10.5 and obtained nine combination groups affected by different dosage levels of each factor. All mice had no considerable health problems but those exposed to 300 mg/kg of VPA had crooked tails (Supplementary Fig. 1) as we described previously^21^. Using these nine groups, we performed the three-chamber sociability interaction test. Vehicle-treated wild-type and *Cntnap2* heterozygous mice, and mice exposed to 100 mg/kg VPA regardless of their genotypes preferred the compartment containing a novel conspecific or its vicinity, whereas *Cntnap*2 KO mice and wild-type (WT) mice exposed to 300 mg/kg VPA had no such preference. However, unexpectedly, the impaired social preference of *Cntnap2* KO mice to a novel conspecific was ameliorated in the offspring of female mice treated with 100 or 300 mg/kg VPA (Fig. 1a–c). To confirm these unexpected findings, we performed the juvenile social play test in the following four groups: *Cntnap2* WT treated with vehicle (WT × Veh), KO treated with vehicle (KO × Veh), WT exposed to 300 mg/kg VPA (WT × VPA), and KO exposed to 300 mg/kg VPA (KO × VPA). In this test, we measured the cumulative social interaction time, including allogrooming, following, pouncing, and sniffing (Fig. 1d). Again, the KO × Veh and WT × VPA groups exhibited significantly shorter social interaction time than the WT × Veh group. However, the social interaction time of the KO × VPA group was similar to that of the WT × Veh group (Fig. 1e; two-way ANOVA, interaction: F(1, 30) = 28.04, *p* < 0.0001; genotype factor: F(1, 30) = 1.56, *p* = 0.2213; VPA factor: F(1, 30) = 0.08728, *p* = 0.7697). Thus, the interaction of *Cntnap2* KO and prenatal VPA exposure alleviates social impairment observed in the respective single-factor models.

### Repetitive self-grooming and hyperactive behaviors are not affected by the interplay of *Cntnap2* KO and prenatal VPA exposure

**Figure 2:**
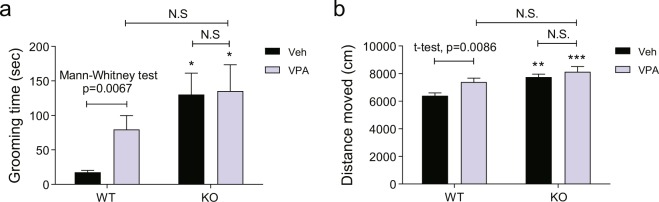
Repetitive self-grooming and hyperactivity of *Cntnap2* KO and VPA-exposed mice are not altered by the interaction of those factors. (**a**) Cumulative time spent on repetitive self-grooming. WT × Veh, n = 10; KO × Veh, n = 12; WT × VPA, n = 8; KO × VPA, n = 10. (**b**) Distance moved in an open-field test. WT × Veh, n = 14; KO × Veh, n = 14; WT × VPA, n = 11; KO × VPA, n = 10. Two-way ANOVA followed by Bonferroni multiple comparison test was performed to determine between-group differences. To compare the indicated groups, Mann-Whitney test was performed in **a** and unpaired *t*-test in **b**. Asterisk (*) shows the results of statistical comparison between WT × Veh and the indicated group; *p < 0.05, **p < 0.01, ***p < 0.001. In all bar graphs, data are the mean ± S.E.M. Abbreviations: WT, *Cntnap2* WT mice; KO, *Cntnap2* KO mice; VPA, treatment with valproic acid (300 mpk) at E10.5; Veh, treatment with phosphate-buffered saline at E10.5.

Next, we investigated the effect of the interaction of *Cntnap2* KO and prenatal exposure to VPA on repetitive behavior, another core symptom of ASD. To this end, we measured cumulative time spent on self-grooming. The *Cntnap2* KO × Veh, WT × VPA, and KO × VPA groups spent significantly more time on self-grooming than did the WT × Veh group (Fig. 2a; Mann-Whitney test: WT × Veh vs. WT × VPA, *p* = 0.067; Bonferroni’s post hoc test: WT × Veh vs. KO × Veh, *p* < 0.05; WT × Veh vs. KO × VPA, *p* < 0.05). However, the self-grooming time in the KO × VPA group was not significantly different from that in the KO × Veh and WT × VPA group (Fig. 2a; two-way ANOVA, interaction: F(1, 36) = 1.021, *p* = 0.3191; genotype factor: F(1, 36) = 8.868, *p* = 0.0052; VPA factor: F(1, 36) = 1.406, *p* = 0.2435).

Hyperactivity is another important symptom observed in the *Cntnap2* KO^19^ and prenatal VPA exposure models^20,22^. To determine the effect of the interaction of these two factors on hyperactivity, we performed the open-field test. The distance moved was significantly longer in the *Cntnap2* KO × Veh and WT × VPA groups than in the WT × Veh group (Fig. 2b; unpaired *t*-test: WT × Veh vs. WT × VPA, *p* = 0.0067; Bonferroni’s post hoc test: WT × Veh vs. KO × Veh, *p* < 0.01). The combination of VPA and *Cntnap2* KO also significantly increased locomotor-activity in comparison with the WT × Veh group. However, the increased locomotor-activity was similar to that of the *Cntnap2* KO × Veh and WT × VPA groups (two-way ANOVA, interaction: F(1, 45) = 1.302, *p* = 0.2599; genotype factor: F(1, 45) = 15.40, *p* = 0.0003; VPA factor: F(1, 45) = 6.542, *p* = 0.0140; Bonferroni’s post hoc test: WT × Veh vs. KO × VPA, *p* < 0.001). Taken together, the above data show that the interaction of *Cntnap2* KO and prenatal VPA exposure does not affect the repetitive and hyperactive behaviors observed in the respective single-factor models.

### Electric shock-induced seizure susceptibility is increased by the interplay of *Cntnap2* KO and prenatal VPA exposure

**Figure 3:**
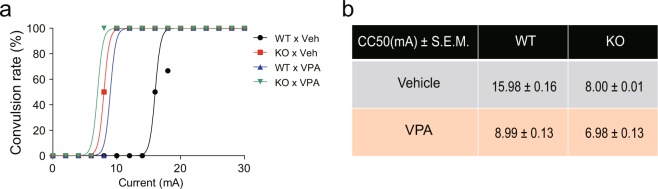
Increased seizure susceptibility in *Cntnap2* KO and VPA-exposed mice is further enhanced by the interaction of these factors. (**a**) Current vs. convulsion rate plot. (**b**) Current that induced convulsions in 50% of the animals was defined as the CC50. Summary of the mean and S.E.M of the CC50. WT × Veh, n = 10; KO × Veh, n = 6; WT × VPA, n = 6; KO × VPA, n = 7. Abbreviations: WT, *Cntnap2* WT mice; KO, *Cntnap2* KO mice; VPA, treatment with valproic acid (300 mpk) at E10.5; Veh, treatment with phosphate-buffered saline at E10.5.

In a previous study, *Cntnap2* KO mice exhibited spontaneous seizures following mild handling stress^19^. VPA-exposed rats also showed increased seizure susceptibility following electric stimulation relative to control rats^20,23^. To examine the effect of the interaction of *Cntnap2* KO and prenatal exposure to VPA on seizure susceptibility, we measured the convulsive threshold determined by the convulsive current 50 (CC50; Fig. 3). The CC50 values were lower in the *Cntnap2* KO × Veh and the WT × VPA groups than in the WT × Veh. Interestingly, the KO × VPA group had lower CC50 values than *Cntnap2* KO × Veh and the WT × VPA groups, suggesting that seizure susceptibility is increased by the interaction of *Cntnap2* KO and prenatal VPA exposure.

### Impaired excitatory transmission in each single-factor model is counterbalanced by the interplay of *Cntnap2* KO and prenatal VPA exposure

**Figure 4:**
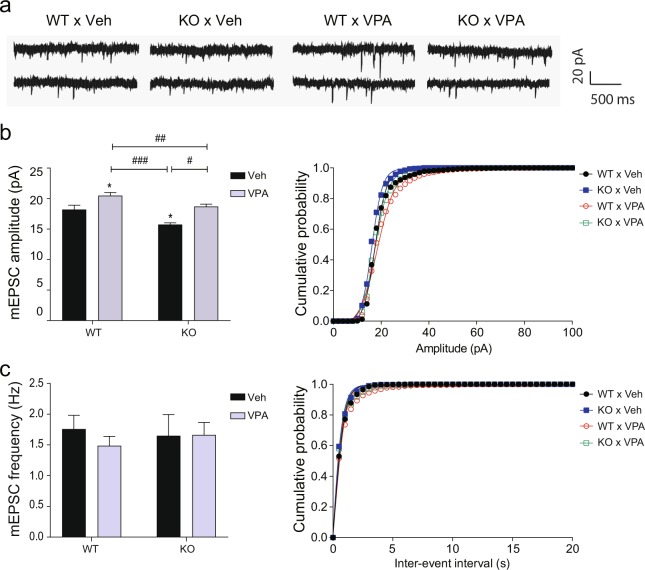
Abnormal mEPSC amplitudes in *Cntnap2* KO and VPA-exposed mice are normalized by the interaction of these factors. Whole-cell patch-clamp recordings in neurons in layer 2/3 of the mPFC. (**a**) Representative mEPSC traces. (**b**) mEPSC amplitudes. (**c**) mEPSC frequencies. Two-way ANOVA followed by Fisher’s LSD test was performed to determine between-group differences. WT × Veh, n = 7; KO × Veh, n = 7; WT × VPA, n = 19; KO × VPA, n = 16. In (**b**) asterisk (*) shows the results of statistical comparison between WT × Veh and the indicated group; *, *p* < 0.05. Sharp (#) shows the results of statistical comparison between the indicated groups; #, *p* < 0.05, ##, *p* < 0.01, ###, *p* < 0.001. In all bar graphs, data are the mean ± S.E.M. Abbreviations: WT, *Cntnap2* WT mice; KO, *Cntnap2* KO mice; VPA, treatment with valproic acid (300 mpk) at E10.5; Veh, treatment with phosphate-buffered saline at E10.5.

**Figure 5:**
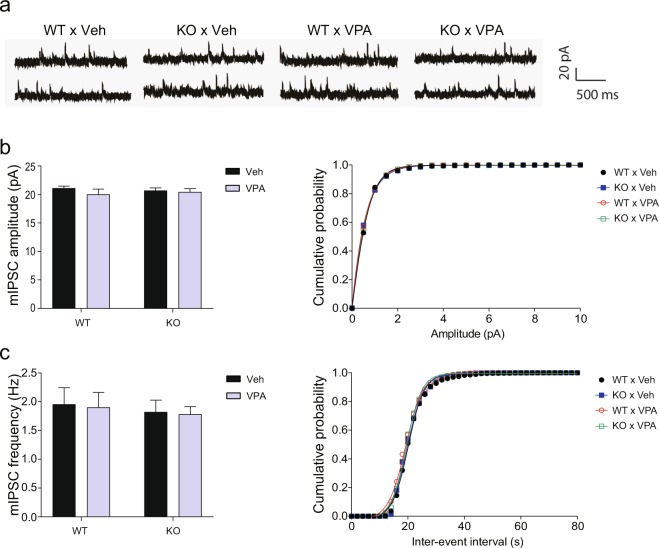
mIPSCs are not affected by *Cntnap2* KO, prenatal VPA exposure, or the interaction of these factors. Whole-cell patch-clamp recordings in neurons in layer 2/3 of the mPFC. (**a**) Representative mIPSC traces. (**b**) mIPSC amplitudes. (**c**) mIPSC frequencies. Two-way ANOVA followed by Fisher’s LSD test was performed to determine significant between-group differences. WT × Veh, n = 9; KO × Veh, n = 9; WT × VPA, n = 10; KO × VPA, n = 11. In all bar graphs, data are the mean ± S.E.M. Abbreviations: WT, *Cntnap2* WT mice; KO, *Cntnap2* KO mice; VPA, treatment with valproic acid (300 mpk) at E10.5; Veh, treatment with phosphate-buffered saline at E10.5.

Proper regulation of neural activity in the mPFC is very important for social behavior^15–17^. In our previous study, *Cntnap2* KO mice and mice prenatally exposed to VPA showed decreased and increased amplitudes of mEPSCs in layer 2/3 pyramidal neurons of the mPFC, respectively, compared to those in the respective control mice^18^. We also observed that the counterbalancing of impaired excitatory transmission using α-amino-3-hydroxy-5-methyl-4-isoxazolepropionic acid (AMPA) modulators specifically improved social behavior in *Cntnap2* KO mice and mice prenatally exposed to VPA^18^. Thus, the improved sociability in the G × E group may be due to neural transmission in the mPFC corrected by the interaction. To examine this possibility, we measured mEPSCs and miniature inhibitory postsynaptic currents (mIPSCs) in layer 2/3 pyramidal neurons of the mPFC (Figs 4 and 5) using whole-cell patch clamping. The mEPSC amplitudes were increased in the WT × VPA groups and reduced in the KO × Veh group in comparison with the WT × Veh group. The KO × VPA group had mEPSCs amplitudes similar to those of the WT × Veh group (Fig. 4b; two-way ANOVA, interaction: F(1, 45) = 0.08325, *p* = 0.7743; genotype factor: F(1, 45) = 12.94, *p* = 0.0008; VPA factor: F(1, 45) = 11.77, *p* = 0.0013). However, there were no significant between-group differences in mEPSC frequencies (Fig. 4c; two-way ANOVA, interaction: F(1, 45) = 0.00004, *p* = 0.9953; genotype factor: F(1, 45) = 0.6577, *p* = 0.4217; VPA factor: F(1, 45) = 0.2836, *p* = 0.5970). There were also no significant between-group differences in the amplitudes of mIPSCs (Fig. 5b; interaction: F(1, 35) = 0.488, *p* = 0.4894; genotype factor: F(1, 35) = 0.0002, *p* = 0.9882; VPA factor: F(1, 35) = 1.153, *p* = 0.2904) and frequencies of mIPSCs (Fig. 5d; interaction: F(1, 35) = 0.0012, *p* = 0.9721; genotype factor: F(1, 35) = 0.3205, *p* = 0.5749; VPA factor: F(1, 35) = 0.0435, *p* = 0.8359). Thus, the interplay of *Cntnap2* KO and prenatal exposure to VPA counterbalances the abnormal excitatory neural transmission in the mPFC of the respective single-factor models, which might contribute to the improved social behaviors in the interaction group.

## Discussion

In this study, we investigated how the interplay between genetic and environmental risk factors affects phenotypic complexity using the *Cntnap2* KO and prenatal exposure to VPA as genetic and environmental factors, respectively. We observed that the interplay between these two factors improved social deficits but increased seizure susceptibility, with no further changes in repetitive self-grooming behaviors or hyperactivity compared to the respective phenotypes of *Cntnap2* KO and VPA mice (Supplementary Table 1). Finally, we also found that the aberrant excitatory neural transmission in the mPFC of the single-factor models was corrected by the interaction of the two factors. Our study provides an important clue to understanding the potential role of gene-environment interaction in the behavioral and pathophysiological spectrum in ASD.

Our results show that behavioral and physiological phenotypes caused by a genetic mutation can be changed by an environmental input. VPA upregulates target gene expression by inhibiting histone deacetylase^24,25^. Thus, the altered gene expression may lead to phenotypic changes in the *Cntnap2* KO mice. For example, our previous studies have shown that prenatal exposure to VPA upregulates Pax6, which may induce differentiation of glutamatergic neurons and thereby enhance synaptic maturation in glutamatergic neurons^21,23^. These changes in glutamatergic neurons caused by prenatal exposure to VPA may alter excitatory neural transmission and behavioral phenotypes in *Cntnap2* KO mice.

Perhaps the most interesting finding in our study is that the interaction of *Cntnap2* KO and prenatal exposure to VPA resulted in normal social behaviors comparable to those of the control group in the three-chamber sociability and juvenile social play tests. Currently, we are not sure how the interaction of the two factors paradoxically recovers the social impairments observed in the respective single-factor models. Our current and previous findings suggest two possibilities. First, we found that the aberrant mEPSC amplitudes in the mPFC region of the single-factor groups were corrected in the KO × VPA group, suggesting that the recovered excitatory neural transmission in the mPFC may be associated with the improved social behaviors in the interaction group. Indeed, neural transmission in the mPFC is very important in regulating social behaviors^15–17^. In our previous study, *Cntnap2* KO mice exhibited an underexpression of glutamatergic receptors and decreased mEPSCs amplitudes in layer 2/3 pyramidal neurons of the mPFC, whereas VPA mice exhibited the opposite pattern^18^. Additionally, a positive allosteric modulator and an antagonist of the AMPA receptor recovered impaired social behaviors without affecting repetitive self-grooming or hyperactivity in *Cntnap*2 KO mice and VPA mice, respectively^18^. Thus, the corrected excitatory neural transmission in the mPFC of the KO × VPA group might underlie the improved social behaviors. However, this possibility should be tested further, since a recent report suggested that social impairments in adult *Cntnap2* KO mice may be induced from abnormal activity of parvalbumin-positive interneurons in the mPFC^26^. This discrepancy may be due to the difference in experimental conditions such as the age of mice. Since we used 4- to 5-week-old mice, whose synaptic structures undergo dynamic changes^27^, the synaptic response might be different from that of adult mice. Thus, further study is required to address this discrepancy. Second, there might be a compensation mechanism in the neural structure of the KO × VPA group. Indeed, a functional magnetic resonance imaging (fMRI) study showed that long-range connectivity in the frontal cortex region was reduced in *CNTNAP2* risk allele carriers^28^. Another fMRI study showed that *Cntnap2* KO mice had reduced local connectivity in the PFC, and the reduction in connectivity showed a significant correlation with impaired social behaviors^29^. On the other hand, increased local connectivity in the mPFC have been suggseted in prenatal VPA exposure model of autism^13,30^. Given that the CNTNAP2 protein has been implicated in synaptic stability^31,32^, the reduced synaptic contacts in *Cntnap2* KO mice may be compensated by the effects of prenatal VPA exposure, which may enhance synaptic maturation in glutamatergic neurons^21^. Thus, the compensation mechanism might contribute to relieving social impairments by recovering abnormal functional connectivity in the PFC. All these possibilities should be tested in further study using more in-depth electrophysiological and imaging approaches.

Another interesting finding in this study is the increased seizure susceptibility of *Cntnap2* KO × VPA mice in comparison with the respective single-factor groups. Seizure susceptibility can be induced by increased excitation or decreased inhibition in any brain region^33^. Since the KO × VPA group exhibited mEPSCs and mIPSCs in the mPFC similar to those of the WT × Veh group, increased excitability in other brain regions may be the reason for the increased seizure susceptibility of the KO × VPA group. For example, in a previous report, *Cntnap2* KO mice had decreased expression of inhibitory neuronal marker proteins such as glutamate decarboxylase (GAD) 67, parvalbumin, calbindin 2, and neuropeptide Y in the somatosensory cortex and striatum, suggesting abnormal inhibitory neuronal function^19^. A whole-cell recording study showed that perisomatic inhibitory neural inputs were decreased in the hippocampal CA1 pyramidal neurons of *Cntnap2* KO mice^34^, suggesting increased excitability in the hippocampus. Another whole-cell recording study showed that *Cntnap2* KO mice displayed reduced excitatory and inhibitory transmission, but the sum of these transmissions resulted in an increased E/I ratio in layer 2/3 of the somatosensory cortex^35^. Moreover, VPA animal models have been suggested to have over-excitation caused not only by increased glutamate receptor expression and transmission^36^ but also by decreased levels of GAD65/67 in the somatosensory cortex^37^. Thus, the interaction of *Cntnap2* KO and prenatal exposure to VPA may increase over-excitability in some brain regions such as the somatosensory cortex, which might contribute to the increased seizure susceptibility in the interaction group.

One confounding result in our study is that the *Cntnap2* KO × VPA interaction did not induce any significant changes on repetitive self-grooming or hyperactivity behaviors, although each factor independently induced these behavioral symptoms. The pathophysiological mechanisms of increased repetitive self-grooming and hyperactivity behaviors in *Cntnap2* KO mice and VPA-exposed models are not clear, yet. In previous studies, the repetitive self-grooming and hyperactivity behaviors were rescued by a dopamine receptor antagonist in *Cntnap2* KO mice^19^ and by N-methyl-D-aspartate (NMDA) receptor blockers in prenatal VPA exposure models^38,39^. These results suggest the involvement of the dopaminergic and glutamatergic systems in these behavioral alterations in *Cntnap2* KO mice and prenatal VPA exposure models, respectively. However, since these two systems are involved in the cortico-basal ganglia-thalamic pathway, which is important for motor coordination and pathophysiology of repetitive and hyperactive behaviors^40,41^, *Cntnap2* KO or prenatal exposure to VPA may be sufficient to fully induce repetitive and hyperactive behaviors, or one of these two factors may be dominant. This may presumably explain why the interplay of *Cntnap2* KO and VPA resulted in no further increase in those behavioral symptoms. Obviously, these possibilities await experimental validation.

Our results suggest several points to consider. First, the interaction of two different risk factors does not always aggravate ASD symptoms but can also alleviate them. Second, an environmental factor can change phenotypes induced by a genetic factor. In addition to prenatal exposure to VPA, other environmental factors such as maternal immune activation^8,42^, advanced paternal age^43,44^, and sex difference^9,45^ may increase the phenotypic complexity of genetic predispositions. Furthermore, an environmental input may rescue impaired neural physiology and autism-like symptoms. Second, although we did not test this here, the effects of an environmental input may depend on its timing. Indeed, VPA is a very well-known teratogen and its effects can be different depending on exposure period^46^. If it is taken during the first trimester period, it greatly increases the risk of craniofacial and limb malformation and neuropsychiatric symptoms such as impaired cognition and autistic behaviors in humans^47^. We have also previously characterized such exposure-period-dependent phenotypic change in animals. We found that animals exposed to VPA during neural tube closure (E12.5^48^ in rats and E10.5^22^ in mice) showed an increased incidence of autism-like symptoms^22,48^. If VPA is given earlier than this critical time point, it causes embryonic lethality. Conversely, if it is given later, VPA does not cause embryonic lethality or autism-like phenotypes^48^. Thus, the timing of exposure may be an important variable in gene-environment interaction studies.

In conclusion, we demonstrated that the interplay of *Cntnap2* KO and prenatal VPA exposure increases the phenotypic complexity by changing the original phenotypes induced by the respective factors. Thus, the complex interaction of multiple factors may explain the behavioral and pathophysiological spectrum in ASD.

## Methods

### Animals

*Cntnap2* KO mice were kindly provided by Dr. Daniel H. Geschwind^19^. The day of checking the vaginal plug was designated as E0 and the day of birth as P0. VPA or PBS was injected subcutaneously at E10.5^22^. From the obtained offspring, 3-week-old male mice were transferred to new cages according to their genotype and treatment (3–6 mice per cage). Genomic DNA was extracted from the tail at P14 and the genotype of each animal was identified using PCR.

Animals were maintained on a standard light-dark cycle (on: 2:00 am; off: 2:00 pm) at 20 °C to 24 °C and relative humidity between 30% and 70%. Animals were given free access to food and water. Animal handling, housing, and treatments, including anesthesia, euthanasia, and administration, were performed in accordance with the Principles of Laboratory Animal Care (NIH publication No. 85-23, revised 1985) and were approved by the Institutional Animal Care and Use Committee of Konkuk University, Korea (KU14142 and KU14143).

### Behavioral studies

Behavioral studies were conducted during the dark cycle. To minimize the unpredictable effects of handling stress, animals were habituated for 3 days to the experimenter’s handling. For this purpose, a mouse was placed on experimenter’s cupped hands and allowed to freely explore for 1 min, once a day. On the day of behavioral tests, mice were acclimated to the behavioral room for 1 h before the tests.

### Three-chamber sociability test

The three-chamber sociability test was conducted as previously reported^18,49^. The task consisted of two sessions. In the first session, the subject mouse was introduced to the middle compartment and habituated for 5 min. After habituation, a new animal (same age, strain, and no previous contact with the subject) was introduced inside a wired cage randomly to either the left or right compartment, while the other wired cage was empty during the 10-min sociability test. Time spent in each compartment and around the cage (within 5 cm from the cage) was measured as stay duration and approach duration, respectively^18^.

### Self-grooming test

The test was conducted as previously reported^18,50^. Before the experiment, each mouse was placed in a polycarbonate cage (20 × 26 × 13 cm) and habituated for 10 min. The cumulative time spent grooming was measured over the next 10 min with observers blinded to the mouse’s group from at least 2 m away from the cage.

### Juvenile social play

The subject mouse was isolated for 30 min in a cage with new bedding (3 cm deep) to stimulate social interaction before the test. A stranger mouse was subsequently introduced to the subject’s cage. The cumulative social interaction time was measured for 10 min. The social interactions included nose-to-nose sniffing, nose-to-anus sniffing, following, allogrooming, and crawling under the partner^51^.

### Electric shock seizure threshold test

Electric shock seizure threshold was measured according to previously reported methods^23^. Briefly, electric shock was applied using an ECT apparatus (Electro Convulsive Therapy, Ugo Basile, Varese, Italy) through ear clips (frequency: 100 pulses/s, pulse width: 0.5 ms, shock duration: 1 s). To minimize the number of animals sacrificed, electric current was increased or decreased stepwise by 2 mA according to the animal’s response to electric shock. A seizure was defined as overt hind limb extension. The convulsive current 50 (CC50) was defined as the current at which 50% of all animals showed seizure and was determined by log current vs. response calculation.

### Open-field test

Exploratory activity in a novel environment was assessed in an open-field box (40 × 40 × 30 cm). Mice were introduced into the center, and the total distance moved in the whole arena and velocity were measured for 20 min using a CCD camera-assisted motion-tracking apparatus and software (EthoVision 3.1, Noldus Information Technology, Leesburg, VA, USA).

### Slice preparation

Slices were prepared from mice aged 4 to 5 weeks. Mice were anesthetized using isoflurane, and the brains were quickly removed and transferred into ice-cold sucrose solution (in mM, 212 sucrose, 3 KCl, 26 NaHCO_3_, 1.25 NaH_2_PO_4_, 7 MgCl_2_, and 10 glucose). Prefrontal cortical coronal slices (350-µm thick) were obtained using a vibratome (Leica VT1200S, Leica Biosystems Inc., Buffalo Grove, IL, USA). Slices were incubated in a submerged holding chamber filled with artificial cerebrospinal fluid (in mM, 118 NaCl, 2.5 KCl, 1 NaH_2_PO_4_, 26.2 NaHCO_3_, 11 glucose, 2 CaCl_2_, and 1 MgCl_2_, oxygenated with 95% O_2_/5% CO_2_) at 35 °C. After recovery for 1 h, brain slices were kept at room temperature.

### Whole-cell patch-clamp recordings

Layer 2/3 pyramidal neurons of the mPFC were visually selected and voltage-clamped at – 60 mV or 0 mV to measure the excitatory or inhibitory transmission, respectively, using an Axoclamp-200B amplifier (Axon Instruments, Union City, CA, USA), filtered at 2 kHz, and sampled at 5 kHz. pClamp software (Version 10.3, Axon Instruments) was used for data acquisition and analysis. The resistance of patch glass pipettes was 2–4 MΩ, and the internal solution used contained, in mM, 115 Cs methanesulphonate, 20 CsCl, 10 HEPES, 2.5 MgCl_2_, 4 Na_2_-ATP, 0.4 Na_2_-GTP, 10 Na-phosphocreatine, and 0.6 EGTA, pH 7.2. The mEPSCs were measured with picrotoxin (50 µM) and tetrodotoxin (1 µM) to block the effects of GABA receptors and depolarization throughout the experiment. The mIPSCs were measured with 6-cyano-7-nitroquinoxaline-2,3-dione (10 µM), AP5 (50 µM), and tetrodotoxin (1 µM) to block the effects of AMPA and NMDA receptors, and depolarization throughout the experiment. Input and series resistance were continuously monitored.

### Statistical analysis

All data are expressed as the mean ± standard error of the mean (S.E.M). Statistical methods are described in each figure legend and in Supplementary Table 2. Briefly, unpaired Student’s *t*-test or Mann-Whitney test was used to determine between-group differences in mean values. To analyze data with two factors, a two-way ANOVA was performed followed by Bonferroni or Fisher’s LSD test. Differences were considered statistically significant when the *P*-value was less than 0.05. All statistical analyses were conducted using GraphPad Prism 5 software (GraphPad Software, La Jolla, CA, USA).

## Supplementary information

Supplementary data

## Data Availability

All data generated or analyzed during this study are included in this published article (and its Supplementary Information File).
