# Supplementary material for: Gene-environment interaction counterbalances social impairment in mouse models of autism
Source: Sci Rep. 2019 Aug 7;9:11490. doi: 10.1038/s41598-019-47680-w (PMC6686010; doi:10.1038/s41598-019-47680-w)
Supplement: Supplementary file 1 — Supplementary data [file 41598_2019_47680_MOESM1_ESM.pdf]

Supplementary information for

Gene-environment interaction counterbalances social impairment in mouse models of autism

Ji-Woon Kim[1], Kwanghoon Park[2], Ri Jin Kang[1], Edson Luck Gonzales[1], Hyun Ah Oh[1], Hana Seung[1], Mee Jung Ko[1], Jae Hoon Cheong[3], ChiHye Chung[2], and Chan Young Shin[1,4,*]

[1]Department of Pharmacology and Department of Advanced Translational Medicine, School of Medicine, Konkuk University, 120 Neungdong-ro, Gwangjin-gu, Seoul 05029, South Korea

[2]Department of Biological Sciences, Konkuk University, 120 Neungdong-ro, Gwangjin-gu, Seoul, 05029, South Korea

[3]Uimyung Research Institute for Neuroscience, Department of Pharmacy, Sahmyook University, 815 Hwarangro, Nowon-gu, Seoul 01795, South Korea

[4]NeuroVenti, Inc. and TriNeuro Inc., 120 Neungdong-ro, Gwangjin-gu, Seoul 05029, South Korea

*Corresponding author

Chan Young Shin, Ph.D.

Department of Pharmacology, School of Medicine, Konkuk University,

1 Hwayang-Dong, Gwangjin-Gu, Seoul 143-701, South Korea

Tel.: +82-2-2030-7834

E-mail: chanyshin@kku.ac.kr

Supplementary Figure 1. Induction of crooked tail by prenatal exposure of VPA.
Crooked tail was observed in mice exposed to 300 mg/kg but not 100 mg/kg of VPA on E10.5

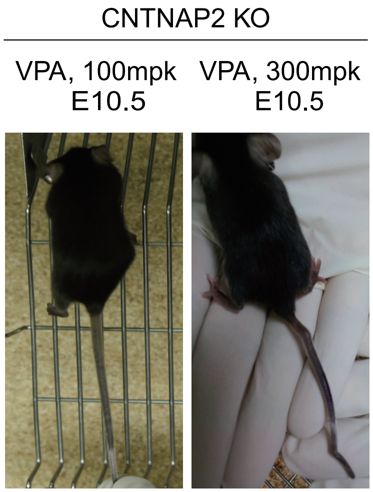

Supplementary Table 1. Summary of behavioral phenotypes in each affected group in comparison with those in WT × Veh group.

	***Cntnap2* KO X Veh**	**VPA(300mpk) X Veh**	**KO X VPA**
**Social**	Impaired	Impaired	Restored<br>(Offsetting)
**Grooming**	Increased	Increased	Increased<br>(No further changes)
**Locomotor**	Increased	Increased	Increased<br>(No further changes)
**Seizure**	susceptible	susceptible	More susceptible<br>(Deterioration)

Supplementary Table 2. Statistical summary

	Group	Side	Number	Mean	STD	SEM	Shapiro-Wilk test	Test	p-value	Significance
**Figure1a**	WT Veh	Stranger	18	276.9	58.22	13.72	**	Mann-Whiteny test	0.0007	***
		Empty	18	209	54.05	12.74	ns			
	HetxVeh	Stranger	35	272.9	89.47	15.12	ns	Unpaired t-test	0.0088	**
		Empty	35	220.3	72.98	12.34	ns			
	KOxVeh	Stranger	25	238.3	76.85	15.37	ns	Unpaired t-test	0.4363	ns
		Empty	25	255.7	79.38	15.88	ns			
	WTx100	Stranger	8	317.7	72.35	25.58	ns	Unpaired t-test	0.0017	**
		Empty	8	180	69.52	24.58	ns			
	Hetx100	Stranger	10	268.8	100.2	31.69	ns	Unpaired t-test	0.9161	ns
		Empty	10	264.1	96.86	30.63	ns			
	KOx100	Stranger	17	316.8	101	24.5	ns	Unpaired t-test	0.0002	***
		Empty	17	192.8	67.02	16.25	ns			
	WTx300	Stranger	11	268.6	38.55	11.62	ns	Unpaired t-test	0.2455	ns
		Empty	11	246.8	46.31	13.96	ns			
	Hetx300	Stranger	19	332	108.5	24.88	ns	Unpaired t-test	0.0001	***
		Empty	19	179.3	109.5	25.11	ns			
	KOx300	Stranger	16	313.1	47.45	11.86	ns	Unpaired t-test	<0.0001	***
		Empty	16	187.6	45.79	11.45	ns			

	Group	Side	Number	Mean	STD	SEM	Shapiro-Wilk test	Test	p-value	Significance
**Figure1b**	WT Veh	Stranger	18	127.7	56.53	13.32	**	Mann-Whiteny test	<0.0001	***
		Empty	18	65.96	36.08	8.504	**			
	HetxVeh	Stranger	35	109.7	47.57	8.041	*	Mann-Whiteny test	<0.0001	***
		Empty	35	70.36	26.34	4.453	ns			
	KOxVeh	Stranger	25	103	38.69	7.738	ns	Unpaired t-test	0.7321	ns
		Empty	25	98.85	46.56	9.312	ns			
	WTx100	Stranger	8	160.2	44.11	15.6	ns	Mann-Whiteny test	0.02078	*
		Empty	8	87.63	68.28	24.14	**			
	Hetx100	Stranger	10	141.7	39.72	12.56	ns	Unpaired t-test	0.0097	**
		Empty	10	90.74	39.05	12.35	ns			
	KOx100	Stranger	17	142.3	75.36	18.28	**	Mann-Whiteny test	0.001	**
		Empty	17	76.45	39.52	9.584	ns			
	WTx300	Stranger	11	146.3	24.9	7.875	ns	Unpaired t-test	0.6126	ns
		Empty	11	138.8	38.86	12.29	ns			
	Hetx300	Stranger	19	182.1	65.53	15.03	ns	Unpaired t-test	<0.0001	***
		Empty	19	71.36	58.39	13.4	ns			
	KOx300	Stranger	16	151.6	46.42	11.61	ns	Unpaired t-test	0.0002	***
		Empty	16	87.98	38.18	9.546	ns			

	Group	Number	Mean	STD	SEM	Two-Way ANOVA test		
**Figure1e**	WT	9	45.14	13.34	4.446	Interaction	F (1, 30) = 28.04	P<0.0001
	KO	8	25.88	15.77	5.576	Geno type factor	F (1, 30) = 1.56	P=0.2213
	WTxVPA	9	21.33	7.171	2.39	VPA factor	F (1, 30) = 0.08728	P=0.7697
	KOxVPA	9	53.22	16.68	5.56			

	Group	Number	Mean	STD	SEM	Two-Way ANOVA test		
**Figure2a**	WT	10	17.5	13.07	4.132	Interaction	F (1, 36) = 1.021	P=0.3191
	KO	12	130.3	107.2	30.96	Geno type factor	F (1, 36) = 8.868	P=0.0052
	WTxVPA	8	79.63	56.75	20.06	VPA factor	F (1, 36) = 1.406	P=0.2435
	KOxVPA	10	135.2	120.9	38.24			

	Group	Number	Mean	STD	SEM	Two-Way ANOVA test		
**Figure2b**	WT	14	6396	777.6	207.8	Interaction	F (1, 45) = 1.302	P=0.2599
	KO	14	7749	803.3	214.7	Geno type factor	F (1, 45) = 15.4	P=0.0003
	WTxVPA	11	7384	943.1	284.3	VPA factor	F (1, 45) = 6.542	P=0.0140
	KOxVPA	10	8127	1217	385			

	Group	Number	CC50	S.E.M.
**Figure3b**	WT	10	17.5	13.07
	KO	6	130.3	107.2
	WTxVPA	6	79.63	56.75
	KOxVPA	7	135.2	120.9

	Group	Number	Mean	STD	SEM	Two-Way ANOVA test		
**Figure4b**	WT	7	18.79	0.7512	0.2839	Interaction	F (1, 45) = 0.08325	P=0.7743
	KO	7	16.67	1.068	0.4038	Geno type factor	F (1, 45) = 12.94	P=0.0008
	WTxVPA	19	20.5	2.153	0.4939	VPA factor	F (1, 45) = 11.77	P=0.0013
	KOxVPA	16	18.7	1.615	0.4038			

	Group	Number	Mean	STD	SEM	Two-Way ANOVA test		
**Figure4c**	WT	7	1.602	0.5825	0.2202	Interaction	F (1, 45) =0.000035	P=0.9953
	KO	7	1.78	0.4841	0.183	Geno type factor	F (1, 45) = 0.6577	P=0.4217
	WTxVPA	19	1.488	0.6642	0.1524	VPA factor	F (1, 45) = 0.2836	P=0.5970
	KOxVPA	16	1.663	0.8082	0.202			

	Group	Number	Mean	STD	SEM	Two-Way ANOVA test		
**Figure5b**	WT	9	21.14	0.9943	0.3314	Interaction	F (1, 35) = 0.488	P=0.4894
	KO	9	20.71	1.338	0.4461	Geno type factor	F (1, 35) = 0.0002	P=0.9882
	WTxVPA	10	20.02	2.953	0.9338	VPA factor	F (1, 35) = 1.153	P=0.2904
	KOxVPA	11	20.47	1.856	0.5596			

	Group	Number	Mean	STD	SEM	Two-Way ANOVA test		
**Figure5c**	WT	9	1.958	0.8597	0.2866	Interaction	F (1, 35) = 0.001243	P=0.9721
	KO	9	1.824	0.6142	0.2047	Geno type factor	F (1, 35) = 0.3205	P=0.5749
	WTxVPA	10	1.904	0.8221	0.26	VPA factor	F (1, 35) = 0.04354	P=0.8359
	KOxVPA	11	1.785	0.4299	0.1296			
